# *Vitamin D3 Receptor* **(** *VDR* **)** Gene rs2228570 **(**Fok1**)** and rs731236 **(**Taq1**)** Variants Are Not Associated with the Risk for Multiple Sclerosis: Results of a New Study and a Meta-Analysis

**DOI:** 10.1371/journal.pone.0065487

**Published:** 2013-06-20

**Authors:** Elena García-Martín, José A. G. Agúndez, Carmen Martínez, Julián Benito-León, Jorge Millán-Pascual, Patricia Calleja, María Díaz-Sánchez, Diana Pisa, Laura Turpín-Fenoll, Hortensia Alonso-Navarro, Lucía Ayuso-Peralta, Dolores Torrecillas, José Francisco Plaza-Nieto, Félix Javier Jiménez-Jiménez

**Affiliations:** 1 Department of Biochemistry and Molecular Biology, University of Extremadura, Cáceres, Spain; 2 Department of Pharmacology, University of Extremadura, Cáceres, Spain; 3 Centro de Investigación Biomédica en Red de Enfermedades Neurodegenerativas, Instituto de Salud Carlos III, Madrid, Spain; 4 Service of Neurology, Hospital Universitario Doce de Octubre, Madrid, Spain; 5 Department of Medicine, University Complutense, Madrid, Spain; 6 Section of Neurology, Hospital La Mancha-Centro, Alcázar de San Juan (Ciudad Real), Spain; 7 Centro de Biología Molecular Severo Ochoa, Facultad de Ciencias, Universidad Autónoma, Madrid, Spain; 8 Department of Medicine-Neurology, Hospital “Príncipe de Asturias,” Universidad de Alcalá, Alcalá de Henares, Madrid, Spain; 9 Section of Neurology, Hospital Universitario del Sureste. Arganda del Rey, Madrid, Spain; Charité University Medicine Berlin, Germany

## Abstract

**Background:**

Some epidemiological, genetic, and experimental data suggest a possible role of vitamin D in the pathogenesis of multiple sclerosis (MS) and in experimental autoimmune encephalomyelitis. Data on the possible contribution of several single nucleotide polymorphisms (SNP) in the *vitamin D receptor* (*VDR*) gene to the risk for MS are controversial. Several studies suggested an interaction between some SNPs in the *VDR* gene and HLADRB1*1501 in the risk for MS.

**Objectives:**

The aim of this study was to investigate a possible influence of the SNPs rs2228570 and rs731236 in the *VDR* gene in the risk for MS. A secondary objective was to address the possible interactions between *VDR* genes and HLADRB1*1501.

**Methods:**

We analyzed the allelic and genotype frequency of *VDR* rs2228570, rs731236, and HLADRB1*1501 (rs3135388) in 303 patients with MS and 310 healthy controls, using *TaqMan* Assays. We also conducted a meta-analysis, that was carried out by using the software Meta-Disc 1.1.1 (http://www.hrc.es/investigacion/metadisc.html; Unit of Clinical Statistics, Hospital Ramón y Cajal, Madrid, Spain). Heterogeneity between studies in terms of degree of association was tested using the Q-statistic.

**Results:**

*VDR* rs2228570 and rs731236 allelic and genotype frequencies did not differ significantly between MS patients and controls, and were unrelated with the age of onset of MS, gender, and course of MS. HLADRB1*1501 showed a high association with the risk of developing MS 4.76(95% C.I.  = 3.14–7.27; p<0.0001). The meta-analysis, after excluding data of one study that was responsible of heterogeneity for rs731236 polymorphism, showed lack of relation of both SNPs with the risk for MS. HLADRB1*1501 showed lack of interaction with *VDR* rs2228570 and rs731236 in increasing MS risk.

**Conclusions:**

These results suggest that *VDR* rs2228570 and rs731236 polymorphisms are not related with the risk for MS, and did not confirm interaction between these *VDR* SNPs and *HLADRB1* in the risk for MS.

## Introduction

Multiple sclerosis (MS) is a chronic inflammatory demyelinating disorder with axonal degeneration affecting the Central Nervous system. The etiology of MS is unknown, but likely multifactorial, with an interplay of genetic, ethnic, geographical and environmental factors (infectious or chemical) [1]–[5]. Some authors proposed that MS is an autoimmune disorder with susceptibility influenced, if not determined, by a relatively small number of genes [1]. Findings from studies on seasonality in MS patients' birth, disease onset and exacerbations, as well as apparent temporal trends in incidence and gender ratio support an influential effect of viruses, metabolic and lifestyle factors on MS risk. Epstein-Barr virus, vitamin D status, and smoking are factors that may explain such epidemiological patterns [4].

A haplotype within the major histocompatibility region is the major risk factor for MS, but despite clear evidence for a genetic component additional risk variants were not identified until the recent advent of genome-wide association studies (GWAS). Until 2010, 11 GWAS have been conducted in MS, and together with follow-up studies these have confirmed 16 loci with genome-wide significance [6], [7]. Many of these common risk variants are located at or near genes with central immunological functions (such as interleukin 2 and 7 receptors, CD58, CD6, CD40, TNFRSF1A and others) and the majority are associated with other autoimmune diseases [6], [7]. A further report of the International Multiple Sclerosis Genetics Consortium, and a Genome Wide metaanalysis identified at least 50 loci related with the risk for MS [8], [9]. However, all loci except HLA showed modest OR in the range of 1.1–1.3 [10]. In particular the association between multiple sclerosis (MS) and the HLA-DRB1*15:01 haplotype has been proven to be strong.

In the last years, investigators have paid attention to a possible role of vitamin D in the etiology of MS [11]:

- Several epidemiological studies suggested association between low 25-hydroxyvitamin D levels and increased MS risk and, on the other hand, MS risk was lower among women whose mothers, while pregnant, had increased vitamin D intake, and among women who had received vitamin D supplements in adolescence.
- Some longitudinal studies showed association between high 25-hydroxyvitamin D levels and lower relapse rates in patients with MS.
- Administration of calcitriol could prevent and slow progression of experimental allergic encephalomyelitis (EAE), and *vitamin D3* could have a beneficial effect on EAE severity in female mice. On the other hand, vitamin D deficiency could reduce EAE severity in mice whose mothers were vitamin D deficient.
- Vitamin D seems to regulate some MS-associated genes, including the HLA-DRB1*15:01 [12].
- *CYP27B1,* a gene related with vitamin D metabolism, has been reported among the new genes associated with MS risk.

Several case-control studies on vitamin D-related genes and MS risk have been conducted [11], [13]. The two most common single nucleotide polymorphisms (SNPs) in the *vitamin D receptor* (*VDR*) gene in Caucasian subjects, rs2228570 (Fok1, formerly rs10735810) and rs731236 (Taq1), have been the most widely studied, with inconsistent results. Two case-control studies showed, respectively, decreased risk for MS related with homozygosis for the minor allele of rs2228570 [14], and with homozygosis for the minor allele of rs731236 [15], while other showed increased risk related with the minor allele of rs731236 [16]. Five case-control studies showed lack of direct association between rs2228570 and MS risk [15]–[19], while other 6 studies showed lack of direct association between rs731236 and MS risk [14], [18], [20]–[23]. Finally, Cox et al. [19] reported a weak evidence of an association between the rs731236C allele and MS when combining data on 1153 trio families and 726 cases and 604 controls, although the study of cases vs. controls showed a modest increase in MS risk related with the minor allele.

**Figure 1:**
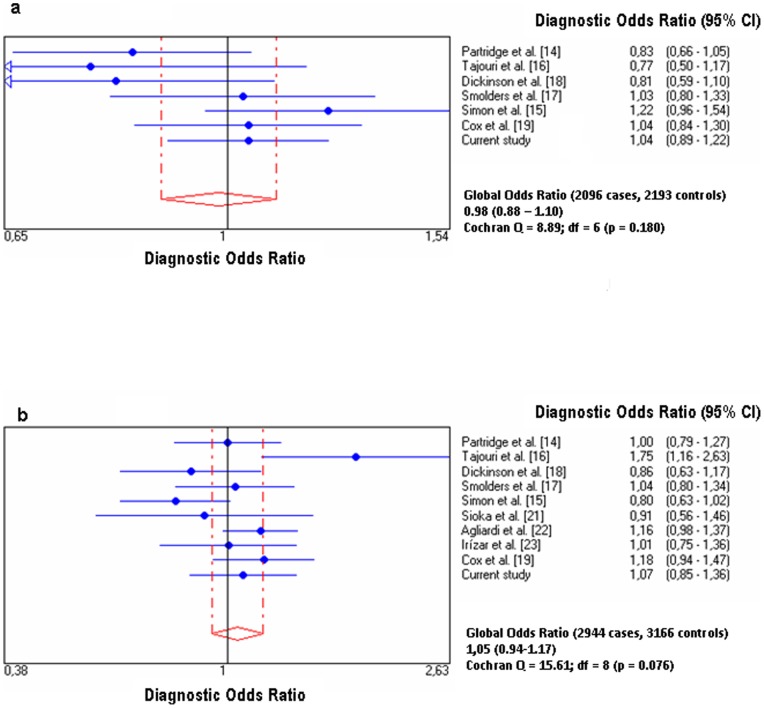
Diagnostic odds ratios and 95% confidence intervals (CI) for each study and for pooled samples of (1–a) rs2228570 (Fok1) and (1–b) rs731236 (Taq1).

Figures 1a and b represent the results of the diagnostic ORs and the 95% confidence intervals(CI) of the studies and the pooled sample, which showed a lack of association of rs2228570 and rs731236 with the risk for MS. Q-statistic showed that studies analyzing both VDR alleles were homogeneous(Q = 8.89, p = 0.180 and Q_ = 15.61, p 0 0.076; respectively).

Simon et al. [15] reported a significant interaction between vitamin D intake and rs2228570 polymorphism (80% of decreased risk of MS for an increase of 400 IU/day of vitamin D in patients homozygous for the minor allele). Agliardi et al. [22] reported decreased MS risk for rs731236TT genotype in HLA-DRB1*15 positive individuals, and Cox et al. [19] reported a trend for increasing risk of MS in subjects who were homozygous for the HLADRB1*1501 (rs3135388) allele in association with rs2228570.

Orton et al. [24], in a family based study (3037 subjects from 739 families; 1360 of which were affected) reported lack of direct association of rs731236 and rs2228570 with MS risk, but they described that HLA-DR15 negative subjects showed over-transmission of the rs2228570T allele. Finally, a meta-analysis of case-control studies showed a trend toward increased risk for MS for the dominant model of the rs731236 SNP and lack of relation of rs2228570 SNP with MS risk [13].

In an attempt to identify additional factors involved in MS susceptibility, we genotyped the SNPs rs731236 and rs2228570 in the *VDR* gene in Spanish Caucasian patients with MS and in healthy subjects. In addition, we have conducted a meta-analysis with the available data of previous reports on the same issue. Despite *VDR* polymorphisms are not mentioned among the possible susceptibility genes in GWAS studies, the possible role of vitamin D in the pathogenesis of MS suggests that *VDR* gene should be a candidate gene for modifying MS risk.

As a secondary objective, to address the possible interactions between *VDR* genes and HLADRB1*1501, as suggested by previous reports [19], [22], [23], we genotyped the SNP HLADRB1*1501 (rs3135388).

## Methods

### Patients and controls

We recruited 303 unrelated Caucasian Spanish patients who fulfilled the McDonald's criteria for definite MS [25] (94 men, 209 women, mean age 43.9±11.4 years, mean age at onset 32.8±10.9 years; 164 relapsing-remitting, 94 secondary progressive, and 45 primary progressive MS; mean ± SD expanded disability status scale or EDSS 4.7±2.2), with no other previous neurological diseases. Recruiting sources were the following: the “Multiple Sclerosis Association of Madrid”; n = 170 cases), the Health Areas of the Hospital La-Mancha-Centro (Alcázar de San Juan, Ciudad Real; n = 70 cases), and University Hospitals “Doce de Octubre” (Madrid, 32 cases), and “Príncipe de Asturias” (Alcalá de Henares, Madrid; n = 31 cases). The control group was composed of 310 healthy unrelated Caucasian Spanish individuals gender and age-matched with the patients (98 men, 212 women; mean age 43.4±11.7 years), most of them students or staff from the University of Extremadura.

### Ethics statement

All the participants were included in the study after giving written informed consent. This study was approved by the Ethics Committee of the University Hospital “Príncipe de Asturias”, University of Alcalá, (Carretera de Alcalá Meco s/n, Alcalá de Henares E28805 Spain). The study was conducted according to the principles expressed in the declaration of Helsinki.

### Genotyping of *VDR* and *HLADRB1* polymorphisms

Genomic DNA was obtained from peripheral leukocytes and purified according to standard procedures. Two common polymorphisms in the *VDR* gene (chromosome 12q12q-14; gene ID: 7421, OMIM 604311) were analyzed. These SNPs were selected on the basis of allele frequencies in Caucasian subjects and were the SNP rs2228570, which causes the amino acid substitution (Met 1 Thr), and the synonymous SNP rs731236, which does not cause amino acid substitution (Ile 352 Ile), but it has been studied with regard to several clinical conditions. Besides the SNPs analyzed, no other nonsynonymous SNPs occur in Caucasian individuals.

*VDR* genotyping was carried out by means of custom TaqMan Assay (Applied Biosciences Hispania, Alcobendas, Madrid, Spain) designed to detect the SNPs rs2228570 (C__12060045_20) and rs731236 (C__2404008_10). HLADRB1*1501 polymorphism was analyzed with the tagging SNP rs3135388 by using a custom TaqMan Assay (Applied Biosciences Hispania, Alcobendas, Madrid, Spain). The detection was carried out by qPCR in an Eppendorf realplex thermocycler by using fluorescent probes. The amplification conditions were as follows: After a denaturation time of 10 min at 96°C, 45 cycles of 92°C 15 sec 60°C 90 sec were carried out and fluorescence was measured at the end of every cycle and at endpoint. All samples were determined by triplicate and genotypes were assigned both, by the gene identification software (RealPlex 2.0, Eppendorf) and by analysis of the reference cycle number for each fluorescence curve, calculated by the use of CalQPlex algorithm (Eppendorf). For technical validation purposes, the amplified fragments for twenty individuals carrying every genotype (TT, CT and CC for rs2228570 and rs731236 and TT, AT and all carriers of the AA genotype (n = 10 in the whole case/control data set) for rs3135388 were sequenced, and in all cases the genotypes fully corresponded with those detected with fluorescent probes.

### Statistical analysis

The intergroup comparison values were calculated by using the chi-square or Fisher tests when appropriate. The 95% confidence intervals were also calculated. The negative predictive value was calculated as d/r2 (d =  number of control individuals with the risk factor absent; r2 =  sum of patients and controls with the risk factor absent). The Hardy-Weinberg equilibrium was confirmed by means of Arlequin software Ver. 2.000.

The sample size was determined from allele frequencies reported for South-European Caucasian individuals as described elsewhere [26], with a genetic model analyzing the frequency the disease gene frequency with a RR value  = 1.5, (P = 0.05). The statistical power for one-tailed and two-tailed associations calculated for the sample size and allele frequencies observed in this study is, 96.3% and 92.4% for the VDR SNP rs2228570, 96.4% and 93.1% for the VDR SNP rs731236 and 53.3% and 40.8% for the HLADRB1*1501 polymorphism rs3135388.

Haplotype reconstruction was performed using the program PHASE v2.1.1. We used the default model for recombination rate variation with 1000 iterations, 500 burn-in iterations and a thinning interval of 1.

Diplotypes were obtained from the combination of haplotypes in the best run (the one that showed the maximum consistency of results across all runs), further details are described elsewhere [27].

### Meta-analysis

All studies that investigated the association of the *VDR* rs2228570 and rs731236 polymorphisms with the development of MS published in any language up to 31 December 2012 and included in the PubMed database were considered. Search strategy used the keywords combinations of “rs2228570 and multiple sclerosis”, “rs731236 and multiple sclerosis”, “vitamin D and multiple sclerosis” and “vitamin D receptor and HLADRB1 and multiple sclerosis”. From each study, information on the number of cases and controls for each *VDR* rs2228570 and rs731236 genotypes was extracted, and allele frequencies were calculated. Due to possible statistical inconsistencies and design differentiations between the studies, the significance of the association between the alleles of *VDR* rs2228570 and *VDR* rs731236 and risk of having MS was tested for each study. All associations were indicated as odds ratios (OR) with the corresponding 95% confidence interval (CI). Based on individual ORs, fixed effects pooled OR and random effects pooled OR were estimated. Meta-analysis of case–control studies was carried out by using the software Meta-DiSc 1.1.1 (http://www.hrc.es/investigacion/metadisc.html; Unit of Clinical Statistics, Hospital Ramón y Cajal, Madrid, Spain) [28]. The global diagnostic OR was calculated with the Mantel-Haenszel [29] method when no heterogeneity was observed. If statistically significant heterogeneity existed, then the global diagnostic OR was calculated with the DerSimonian-Laird method [30]. Heterogeneity between studies in terms of degree of association was tested using the Q-statistic, which is a weighted sum of squares of the deviations of individual study OR estimates from the overall estimate. When ORs are homogeneous, Q follows a chi-squared distribution with r-1 (r is the number of studies) degrees of freedom (d.f.). If P>0.10, then heterogeneity was considered significant.

In order to ensure the rigor of this current meta-analysis, we designed and reported it according to the Preferred Reporting Items for Systematic Reviews and Meta-analyses (PRISMA) statement and the checklist is shown in Table S1 (http://www.prisma-statement.org).

## Results

### Current study

The frequencies of *VDR* rs2228570 (Fok1) and rs731236 (Taq1) genotypes and allelic variants in patients with MS did not differ from those of controls (Table 1). The genotype and allele frequencies between MS patients and healthy subjects were in Hardy-Weinberg's equilibrium. Mean age at onset of MS did not differ significantly between patients carrying *VDR* rs2228570 (Fok1) A/A (mean ± SD  = 33.0±11.5 years), A/G (mean ± SD  = 32.4±9.8 years) and G/G genotypes (mean ± SD  = 38.5±24.7 years; p =  n.s. for the comparison of carriers vs non-carriers of variant alleles), and between patients with genotypes *VDR* rs731236 (Taq1) T/T (mean ± SD  = 32.5±11.5 years), C/T (mean ± SD  = 32.6±10,6 years) and C/C (mean ± SD  = 35.6±10.9 years; p =  n.s. for the comparison of carriers vs non-carriers of variant alleles).

**Table 1:** *Vitamin D3 Receptor* (*VDR*) genotype and allelic variants of patients with multiple sclerosis (MS) and healthy volunteers.

	GENOTYPES						ALLELES			
	*VDR rs2228570 *(*Fok1*)*A/A*	*VDR rs2228570 *(*Fok1*) *A/G*	*VDR rs2228570 *(*Fok1*) *G/G*	*VDR rs731236 *(*Taq1*) *T/T*	*VDR rs731236 *(*Taq1*) *C/T*	*VDR rs731236 * (*Taq1*) *C/C*	*VDR rs2228570 *(*Fok1*) *A*	*VDR rs2228570 * (*Fok1*) *G*	*VDR rs731236 * (*Taq1*) *T*	*VDR rs731236 * (*Taq1*) *C*
**MS PATIENTS** (**N = 303, 606 alleles**)	32(10.6; 7.1–14.0)	141(46.5; 40.9–52.2)	130(42.9; 37.3–48.5)	129(42.6; 37.0–48.1)	129(42.6; 37.0–48.1)	45(14.9; 10.8–18.9)	205(33.8; 30.1–37.6)	401(66.2; 62.4–69.9)	387(63.9; 60.0–67.7)	219(36.1; 32.3–40.0)
**CONTROLS** (**N = 310, 620 alleles**)	42(13.5; 9.7–17.4)	124(40.0; 34.5–45.5)	144(46.5; 40.9–52.0)	131(42.3; 36.8–47.8)	144(46.5; 40.9–52.0)	35(11.3; 7.8–14.8)	208(33.5; 29.8–37.3)	412(66.5; 62.7–70.2)	406(65.5; 61.7–69.2)	214(34.5; 30.8–38.3)
**Intergroup comparison values OR **(**95% CI**)**; P. NPV**(**95% CI**)	0.75(0.45–1.26), 0.257. 0.50(0.48–0.51)	1.31(0.94–1.82), 0.103. 0.53(0.50–0.57)	0.87(0.62–1.21), 0.378. 0.49(0.45–0.53)	1.01(0.73–1.41), 0.937. 0.51(0.47–0.54)	0.86(0.61–1.19), 0.335. 0.49(0.45–0.53)	1.37(0.83–2.26), 0.191. 0.52(0.50–0.53)	1.01(0.79–1.29), 0.917. 0.51(0.49–0.53)	0.99(0.77–1.26), 0.917. 0.50(0.46–0.54)	0.93(0.73–1.19), 0.552. 0.49(0.46–0.53)	1.07(0.84–1.37), 0.552. 0.51(0.49–0.53)
**MS WOMEN** (**N = 209, 418 alleles**)	21(10.0; 6.0–14.1)	97(46.4; 39.7–53.2)	91(43.5; 36.8–50.3)	85(40.7; 34.0–47.3)	95(45.5; 38.7–52.2)	29(13.9; 9.2–18.6)	139(33.3; 28.7–37.8)	279(66.7; 62.2–71.3)	265(63.4; 58.8–68.0)	153(36.6; 32.0–41.2)
**CONTROL WOMEN** (**N = 212, 424 alleles**)	25(11.8; 7.5–16.1)	89(42.0; 35.3–48.6)	98(46.2; 39.5–52.9)	89(42.0; 35.3–48.6)	99(46.7; 40.0–53.4)	24(11.3; 7.1–15.6)	139(32.8; 28.3–37.3)	285(67.2; 62.7–71.7)	277(65.3; 60.8–69.9)	147(34.7; 30.1–39.2)
**Intergroup comparison values OR** (**95% CI**)**; P. NPV** (**95% CI**)	0.84(0.43–1.61), 0.567. 0.50–0.48–0.52	1.20(0.80–1.79), 0.361. 0.52(0.48–0.57)	0.90(0.60–1.34), 0.580. 0.49(0.45–0.54)	0.95(0.63–1.42), 0.785. 0.50(0.46–0.54)	0.95(0.64–1.42), 0.798. 0.50(0.45–0.54)	1.26(0.68–2.34), 0.430. 0.51(0.49–0.53)	1.02(0.76–1.38), 0.885. 0.51(0.48–0.53)	0.98(0.73–1.32), 0.885. 0.50(0.45–0.55)	0.92(0.69–1.23), 0.558. 0.49(0.44–0.54)	1.09(0.81–1.46), 0.558. 0.51(0.49–0.54)
**MS MEN** (**N = 94, 188 alleles**)	11(11.7; 5.2–18.2)	44(46.8; 36.7–56.9)	39(41.5; 31.5–51.4)	44(46.8; 36.7–56.9)	34(36.2; 26.5–45.9)	16(17.0; 9.4–24.6)	66(35.1; 28.3–41.9)	122(64.9; 58.1–71.7)	122(64.9; 58.1–71.7)	66(35.1; 28.3–41.9)
**CONTROL MEN **(**N = 98, 196 alleles**)	17(17.3; 9.9–24.8)	35(35.7; 26.2–45.2)	46(46.9; 37.1–56.8)	42(42.9; 33.1–52.7)	45(45.9; 36.1–55.8)	11(11.2; 5.0–17.5)	69(35.2; 28.5–41.9)	127(64.8; 58.1–71.5)	129(65.8; 59.2–72.5)	67(34.2; 27.5–40.8)
**Intergroup comparison values OR** (**95% CI**)**; P. NPV** (**95% CI**)	0.63(0.26–1.53), 0.269. 0.49(0.47–0.53)	1.59(0.85–2.95), 0.119. 0.56(0.49–0.62)	0.80(0.44–1.48), 0.448. 0.49(0.42–0.55)	1.17(0.64–2.16), 0.583. 0.53(0.46–0.60)	0.67(0.36–1.24), 0.171. 0.47(0.41–0.53)	1.62(0.66–4.01), 0.249. 0.53(0.50–0.56)	1.00(0.64–1.55), 0.984. 0.65(0.60–0.70)	1.00(0.65–1.56), 0.984. 0.51(0.44–0.58)	0.96(0.62–1.49), 0.850. 0.50(0.43–0.58)	1.04(0.67–1.62), 0.850. 0.51(0.48–0.55)

The values in each cell represent: number (percentage; 95% confidence intervals CI). NPV negative predictive value.

The distribution of rs2228570 and rs731236 allelic and genotype frequencies were not influenced by gender (table 1). The distribution of the rs2228570 and rs731236 genotype and allelic frequencies did not differ among the MS phenotypes such as “relapsing-remiting”, “primary progressive”, and “secondary progressive” evolutive types of MS, and between each type with controls (table 2).

**Table 2:** *VDR* genotypes and allelic variants in patients with MS, and relation with the evolutive type of MS.

	GENOTYPES						ALLELES			
	*VDR rs2228570 *(*Fok1*)*A/A*	*VDR rs2228570 *(*Fok1*) *A/G*	*VDR rs2228570 *(*Fok1*) *G/G*	*VDR rs731236 *(*Taq1*) *T/T*	*VDR rs731236 *(*Taq1*) *C/T*	*VDR rs731236 *(*Taq1*) *C/C*	*VDR rs2228570 *(*Fok1*) *A*	*VDR rs2228570 *(*Fok1*) *G*	*VDR rs731236 *(*Taq1*) *T*	*VDR rs731236 *(*Taq1*) *C*
**RELAPSING-REMITTING MS (N = 164; 328 alleles)**	16(9.8; 5.2–14.3)	74(45.1; 37.5–52.7)	74(45.1; 37.5–52.7)	78(47.6; 39.9–55.2)	59(36.0; 28.6–43.3)	27(16.5; 10.8–22.1)	106(32.3; 27.3–37.4)	222(67.7; 62.6–72.7)	215(65.5; 60.4–70.7)	113(34.5; 29.3–39.6)
**Comparison with control subjects. OR (95% CI); P. NPV (95% CI)**	0.69(0.36–1.32); 0.231. 0.64(0.63–0.66)	1.23(0.83–1.84); 0.283. 0.67(0.64–0.71)	0.95(0.64–1.41); 0.783. 0.65(0.61–0.69)	1.24(0.83–1.85); 0.269. 0.68(0.64–0.72)	0.65(0.43–0.97); 0.029. 0.61(0.58–0.65)	1.55(0.88–2.75); 0.112. 0.67(0.65–0.69)	0.95(0.70–1.27); 0.702. 0.65(0.63–0.67)	1.06(0.79–1.42); 0.702. 0.66(0.62–0.71)	1.00(0.75–1.34); 0.984. 0.65(0.61–0.70)	1.00(0.75–1.33); 0.984. 0.65(0.63–0.68)
**SECONDARY PROGRESSIVE MS (N = 94; 188 alleles)**	10(10.6; 4.4–16.9)	51(54.3; 44.2–64.3)	33(35.1; 25.5–44.8)	35(37.2; 27.5–47.0)	46(48.9; 38.8–59.0)	13(13.8; 6.9–20.8)	71(37.8; 30.8–44.7)	117(62.2; 55.3–69.2)	116(61.7; 54.8–68.7)	72(38.3; 31.3–45.2)
**Comparison with control subjects. OR (95% CI); P. NPV (95% CI)**	0.76(0.34–1.65); 0.461. 0.76(0.75–0.78)	1.78(1.09–2.91); 0.015. 0.81(0.77–0.85)	0.62(0.38–1.03); 0.052. 0.73(0.70–0.77)	0.81(0.49–1.34); 0.386. 0.75(0.72–0.79)	1.11(0.68–1.80); 0.673. 0.78(0.74–0.82)	1.26(0.60–2.61); 0.506. 0.77(0.76–0.79)	1.20(0.84–1.71); 0.287. 0.78(0.76–0.80)	0.83(0.59–1.18); 0.287. 0.75(0.70–0.79)	0.85(0.60–1.21); 0.342. 0.75(0.71–0.79)	1.18(0.83–1.67); 0.342. 0.78(0.76–0.80)
**PRIMARY PROGRESSIVE MS (N = 45; 90 alleles)**	6(13.3; 3.4–23.3)	16(35.6; 21.6–49.5)	23(51.1; 36.5–65.7)	16(35.6; 21.6–49.5)	24(53.3; 38.8–67.9)	5(11.1; 1.9–20.3)	28(31.1; 21.5–40.7)	62(68.9; 59.3–78.5)	56(62.2; 52.2–72.2)	34(37.8; 27.8–47.8)
**Comparison with control subjects. OR (95% CI); P. NPV (95% CI)**	0.98(0.35–2.61); 0.969. 0.87(0.86–0.89)	0.83(0.41–1.66); 0.569. 0.87(0.84–0.90)	1.21(0.62–2.36); 0.559. 0.88(0.85–0.92)	0.75(0.37–1.51); 0.394. 0.86(0.83–0.89)	1.32(0.67–2.58); 0.388. 0.89(0.85–0.92)	0.98(0.32–2.82); 0.972. 0.87(0.86–0.89)	0.90(0.54–1.48); 0.647. 0.87(0.85–0.89)	1.12(0.68–1.85); 0.647. 0.88(0.84–0.92)	0.87(0.54–1.41); 0.544. 0.86(0.83–0.90)	1.15(0.71–1.86); 0.544. 0.88(0.86–0.90)
**ALL CONTROLS (N = 310, 620 alleles)**	42(13.5; 9.7–17.4)	124(40.0; 34.5–45.5)	144(46.5; 40.9–52.0)	131(42.3; 36.8–47.8)	144(46.5; 40.9–52.0)	35(11.3; 7.8–14.8)	208(33.5; 29.8–37.3)	412(66.5; 62.7–70.2)	406(65.5; 61.7–69.2)	214(34.5; 30.8–38.3)

The values in each cell represent: number (percentage; 95% confidence intervals). NPV negative predictive value.

We also examined interactions between the VDR SNPs with the tagging SNP rs3135388 for the HLA-DRB*1501 locus containing a highly conserved vitamin D responsive element [19]. Table 3 shows that the rs3135388 SNP has a high association with the risk of developing MS, with an OR for carriers of variant alleles equal to 4.76(95% C.I.  = 3.14–7.27; p<0.0001), with a significant gene dose effect according the Armitage's test for trend with the number of variant alleles (Chi-square  = 67.61; p<0.0001). Tables 3 and 4 show that the association of the SNP rs3135388 with the risk of developing MS, is independent of the gender and of the evolutive type of the disease. The rs3135388 genotype and allele frequencies between MS patients and healthy subjects were in Hardy-Weinberg's equilibrium. Mean age at onset of MS did not differ significantly between patients carrying rs3135388 T/T (mean ± SD  = 33.2±11.3 years), A/T (mean ± SD  = 32.3±10.7 years) and A/A genotypes (mean ± SD  = 31.3±4.0 years; p =  n.s. for the comparison of carriers vs non-carriers of variant alleles). Although the sample size is insufficient to detect weak gene-gene interactions with a high statistical power, we observed no significant differences in gene-gene combination frequencies (Table 5) when we analyzed the interaction between the HLA-DRB1*1501 tagging SNP (rs3135388), and the VDR gene polymorphisms, as described elsewhere [19].

**Table 3:** HLADRB1*1501 (rs3135388) genotype and allelic variants of patients with multiple sclerosis (MS) and healthy volunteers.

	GENOTYPES			ALLELES	
	*rs3135388 T/T*	*rs3135388 T/A*	*rs3135388 A/A*	*rs3135388 T*	*rs3135388 A*
**MS PATIENTS (N = 303, 606 alleles)**	184(60.7; 55.2–66.2)	110(36.3; 30.9–41.7)	9(3.0; 1.1–4.9)	478(78.9; 75.6–82.1)	128(21.1; 17.9–24.4)
**CONTROLS (N = 310, 620 alleles)**	278(89.7; 86.3–93.1)	31(10.0; 6.7–13.3)	1(0.3; 0.−3–1.0)	587(94.7; 92.9–96.4)	33(5.3; 3.6–7.1)
**Intergroup comparison values OR (95% CI); P.**	0.18(0.11–0.28); <0.0001.	5.13(3.24–8.16); <0.0001.	9.46(1.22–200.64); 0.010	0.21(0.14–0.32); <0.0001.	4.76(3.141–7.27); <0.0001.
**MS WOMEN (N = 209, 418 alleles)**	123(58.9; 52.2–65.5)	82(39.2; 32.6–45.9)	4(1.9; 0.1–3.8)	328(78.5; 74.5–82.4)	90(21.5; 17.6–25.5)
**CONTROL WOMEN (N = 212, 424 alleles)**	190(89.6; 85.5–93.7)	21(9.9; 5.9–13.9)	1(0.5; 0.−5–1.4)	401(94.6; 92.4–96.7)	23(5.4; 3.3–7.6)
**Intergroup comparison values OR (95% CI); P.**	0.17(0.10–0.29); <0.0001.	5.87(3.36–10.33); <0.0001	4.11(0.43–97.56); 0.173	0.21(0.13–0.35); <0.0001	4.78(2.98–7.97); <0.0001
**MS MEN (N = 94, 188 alleles)**	61(64.9; 55.2–74.5)	28(29.8; 20.5–39.0)	5(5.3; 0.8–9.9)	150(79.8; 74.0–85.5)	38(20.2; 14.5–26.0)
**CONTROL MEN (N = 98, 196 alleles)**	88(89.8; 83.8–95.8)	10(10.2; 4.2–16.2)	0(0.0; 0.0–0.0)	186(94.9; 91.8–98.0)	10(5.1; 2.0–8.2)
**Intergroup comparison values OR (95% CI); P.**	0.21(0.09–0.49); <0.0001	3.73(0.16–8.89); 0.001	–; 0.021	0.21(0.10–0.46); <0.0001	4.71(2.17–10.47); <0.0001

The values in each cell represent: number (percentage; 95% confidence intervals CI).

**Table 4:** HLADRB1*1501 (rs3135388) genotypes and allelic variants in patients with MS, and relation with the evolutive type of MS.

	GENOTYPES			ALLELES	
	*rs3135388 T/T*	*rs3135388 T/A*	*rs3135388 A/A*	*rs3135388 T*	*rs3135388 A*
**RELAPSING-REMITTING MS (N = 164; 328 alleles)**	98(59.8; 52.3–67.3)	62(37.8; 30.4–45.2)	4(2.4; 0.1–4.8)	258(78.7; 74.2–83.1)	70(21.3; 16.9–25.8)
**Comparison with control subjects. OR (95% CI); P.**	0.17(0.10–0.28); <0.0001	5.47(3.28–9.18);**<0.0001	7.73(0.81–183.04); 0.032	0.21(0.13–0.33);**<0.0001	4.83(3.05–7.67);**<0.0001
**SECONDARY PROGRESSIVE MS (N = 94; 188 alleles)**	58(61.7; 51.9–71.5)	33(35.1; 25.5–44.8)	3(3.2; 0.−4–6.7)	149(79.3; 73.5–85.1)	39(20.7; 14.9–26.5)
**Comparison with control subjects. OR (95% CI); P.**	0.19(0.10–0.33); <0.0001	4.87(2.67–8.89);**<0.0001	10.19(0.93–257.32); 0.014	0.22(0.13–0.36);**<0.0001	4.66(2.75–7.88);**<0.0001
**PRIMARY PROGRESSIVE MS (N = 45; 90 alleles)**	28(62.2; 48.1–76.4)	15(33.3; 19.6–47.1)	2(4.4; −1.–6–10.5)	71(78.9; 70.5–87.3)	19(21.1; 12.7–29.5)
**Comparison with control subjects. OR (95% CI); P.**	0.19(0.89–0.41); <0.0001	4.50(2.05–9.82);**<0.0001	14.37(0.99–409.67); 0.005	0.21(0.11–0.41);**<0.0001	4.76(2.46–9.18);**<0.0001
**ALL CONTROLS (N = 310, 620 alleles)**	278(89.7; 86.3–93.1)	31(10.0; 6.7–13.3)	1(0.3; 0.−3–1.0)	587(94.7; 92.9–96.4)	33(5.3; 3.6–7.1)

The values in each cell represent: number (percentage; 95% confidence intervals).

**Table 5:** Gene-gene interaction between the HLA-DRB1*1501 tagging SNP (rs3135388), and the VDR gene polymorphisms in patients with MS.

rs3135388 (HLADRB1*1501)	rs2228570 (*VDR* Fok1)	*Patients Frequency: N*(*%; 95% CI*)	*Controls Frequency: N*(*%; 95% CI*)	Intergroup comparison values. OR (95% CI); P. NPV (95% CI)
**T/T**	**A/A**	22(12.0; 7.3–16.7)	37(13.4; 9.4–17.4)	0.89(0.49–1.61); 0.675. 0.60(0.58–0.62)
**T/T**	**A/G**	83(45.4; 38.1–52.6)	111(40.1; 34.3–45.8)	1.24(0.84–1.84); 0.262. 0.62(0.58–0.66)
**T/T**	**G/G**	78(42.6; 35.5–49.8)	129(46.6; 40.7–52.4)	0.85(0.58–1.26); 0.405. 0.59(0.54–0.63)
**A/T**	**A/A**	8(7.3; 2.4–12.1)	4(12.9; 1.1–24.7)	0.53(0.13–2.28); 0.323. 0.21(0.18–0.23)51
**A/T**	**A/G**	51(46.4; 37.0–55.7)	12(38.7; 21.6–55.9)	1.36(0.57–3.34); 0.451. 0.24(0.18–0.30)
**A/T**	**G/G**	51(46.4; 37.0–55.7)	15(48.4; 30.8–66.0)	0.92(0.39–2.20); 0.842. 0.21(0.15–0.28)
**A/A**	**A/A**	2(22.2; −4.−9–49.4)	0(0.0; 0.0–0.0)	–; 0.617. 0.13(0.01–0.13)
**A/A**	**A/G**	6(66.7; 35.9–97.5)	1(100.0; 100.0–100.0)	–; 0.513. 0.0(0.0–0.32)
**A/A**	**G/G**	1(11.1; −9.−4–31.6)	0(0.0; 0.0–0.0)	–; 0.739.(0.11(0.10–0.11)

rs3135388 (HLADRB1*1501)	*rs731236* (*VDR Taq1*)	*Frequency: N*(*%; 95% CI*)	*Controls Frequency: N*(*%; 95% CI*)	Intergroup comparison values. OR (95% CI); P. NPV (95% CI)
**T/T**	**T/T**	79(43.2; 36.0–50.3)	118(42.6; 36.8–48.4)	1.02(0.69–1.52); 0.904. 0.61(0.56–0.65)
**T/T**	**C/T**	81(44.3; 37.1–51.5)	129(46.6; 40.7–52.4)	0.91(0.62–1.35); 0.627. 0.59(0.55–0.64)
**T/T**	**C/C**	23(12.6; 7.8–17.4)	30(10.8; 7.2–14.5)	1.18(0.64–2.19); 0.568. 0.61(0.59–0.62)
**A/T**	**T/T**	44(40.0; 30.8–49.2)	13(41.9; 24.6–59.3)	1.4(0.63–3.30); 0.351. 0.30(0.24–0.35)
**A/T**	**C/T**	46(41.8; 32.6–51.0)	15(48.4; 30.8–66.0)	0.77(0.32–1.84); 0.516. 0.20(0.14–0.26)
**A/T**	**C/C**	20(18.2; 11.0–25.4)	3(9.7; 0.−7–20.1)	2.07(0.53–9.50); 0.259. 0.24(0.20–0.26)
**A/A**	**T/T**	6(66.7; 35.9–97.5)	1(100.0; 100.0–100.0)	–; 0.513. 0.0(0.0–0.32)
**A/A**	**C/T**	2(22.2; −4.−9–49.4)	0(0.0; 0.0–0.0)	–; 0.617. 0.13(0.01–0.13)
**A/A**	**C/C**	1(11.1; −9.−4–31.6)	0(0.0; 0.0–0.0)	–; 0.739. 0.11(0.01–0.11)

NPV negative predictive value.

Combinations for all genotypes were analyzed in 302 MS patients and 309 controls. The frequencies shown correspond to the frequencies of a particular combination of VDR genotype within every rs3135388 (HLADRB1*1501) genotype category.

### Metaanalysis

We identified, including the current study, 7 studies analyzing the influence of the SNP rs2228570 (2096 MS patients and 2193 controls), and 10 studies on the influence of the SNP rs731236 (2944 MS patients and 3166 controls) and the risk for MS. Data on these studies, including the genotype and allelic frequencies of the rs2228570 and rs731236, are showed in Tables 6 and 7, respectively.

**Table 6:** Case-control studies on *VDR* rs2228570 (Fok1) and risk for MS (NA =  data not available).

AUTHORS [REF]	GROUP (N)	GENOTYPE FF	GENOTYPE Ff	GENOTYPE ff	ALLELE F	ALLELE f
Partridge et al. [14]	MS(406)	155(0.382)	196(0.483)	55(0.135)	506(0.623)	306(0.377)
	Controls(234)	83(0.355)	105(0.449)	46(0.196)	271(0.579)	197(0.421)
	OR(95% CI), p	1.12(0.79–1.60), 0.495	1.15(0.82–1.61), 0.406	0.64(0.41–1.01), 0.041	1.20(0.95–1.53), 0.120	0.83(0.66–1.06), 0.120
Tajouri et al. [16]	MS(98)	47(0.480)	40(0.408)	11(0.112)	134(0.684)	62(0.316)
	Controls(93)	34(0.365)	48(0.516)	11(0.118)	116(0.624)	70(0.376)
	OR(95% CI), p	1.60(0.86–2.98), 0.112	0.65(0.35–1.19), 0.136	0.94(0.36–2.49), 0.896	1.30(0.84–2.04), 0.218	0.77(0.49–1.20), 0.218
Dickinson et al. [18]	MS(136)	58(0.426)	61(0.449)	17(0.125)	177(0.651)	95(0.349)
	Controls(235)	86(0.366)	110(0.468)	39(0.166)	282(0.600)	188(0.400)
	OR(95% CI), p	1.29(0.82–2.03), 0.250	0.92(0.59–1.44), 0.716	0.72(0.37–1.38), 0.289	1.24(0.90–1.72), 0.171	0.81(0.58–1.11), 0.171
Smolders et al. [17]	MS(212)	79(0.373)	103(0.486)	30(0.141)	261(0.616)	163(0.384)
	Controls(289)	113(0.391)	134(0.464)	42(0.145)	360(0.623)	218(0.377)
	OR(95% CI), p	0.93(0.63–1.36), 0.676	1.09(0.75–1.58), 0.624	0.97(0.57–1.66), 0.904	0.97(0.74–1.27), 0.815	1.03(0.79–1.35), 0.815
Simon et al. [15]	MS(214)	77(0.360)	96(0.449)	41(0.191)	250(0.584)	178(0.416)
	Controls(428)	176(0.411)	188(0.439)	64(0.150)	540(0.631)	316(0.369)
	OR(95% CI), p	0.81(0.57–1.15), 0.209	1.04(0.74–1.47), 0.822	1.35(0.86–2.12), 0.175	0.82(0.64–1.05), 0.105	1.22(0.95–1.56), 0.105
Cox et al. [19]	MS(727)	NA	NA	NA	886(0.609)	568(0.391)
	Controls(604)	NA	NA	NA	748(0.619)	460(0.381)
	OR(95% CI), p	–	–	–	0.96(0.82–1.13), 0.603	1.04(0.89–1.22), 0.603
García-Martín et al.(Current study)	MS(303)	130(0.429)	141(0.465)	32(0.106)	401(0.662)	205(0.338)
	Controls(310)	144(0.465)	124(0.400)	42(0.135)	412(0.665)	208(0.335)
	OR(95% CI), p	0.87(0.62–1.21), 0.378	1.31(0.94–1.82), 0.103	0.75(0.45–1.26), 0.257	0.99(0.77–1.26), 0.917	1.01(0.79–1.29), 0.917
TOTAL SERIES	MS(2096)	546/1369(0.399)	637/1369(0.465)	186/1369(0.136)	2615(0.624)	1577(0.376)
	Controls(2193)	636/1589(0.400)	709/1589(0.446)	244/1589(0.153)	2729(0.622)	1657(0.377)
	OR (95% CI), p	0.99(0.86–1.16), 0.937	1.08(0.93–1.25), 0.298	0.87(0.70–1.07), 0.174	1.01(0.92–1.10), 0.879	0.99(0.91–1.09), 0.879

**Table 7:** Case-control studies on *VDR* rs731236 (Taq1) and risk for MS (NA =  data not available).

AUTHORS [REF]	GROUP (N)	GENOTYPE TT	GENOTYPE Tt	GENOTYPE tt	ALLELE T	ALLELE t
Partridge et al. [14]	MS(402)	140(0.348)	203(0.505)	59(0.147)	483(0.601)	321(0.399)
	Controls(231)	86(0.372)	106(0.459)	39(0.169)	278(0.602)	184(0.398)
	OR(95% CI), p	0.90(0.63–1.28), 0.544	1.20(0.86–1.69), 0.264	0.85(0.53–1.35), 0.460	1.00(0.78–1.27), 0.973	1.00(0.79–1.28), 0.973
Tajouri et al. [16]	MS(104)	27(0.260)	57(0.548)	20(0.192)	111(0.534)	97(0.466)
	Controls(93)	42(0.452)	40(0.430)	11(0.118)	124(0.667)	62(0.333)
	OR(95% CI), p	0.43(0.22–0.81), 0.005	1.61(0.88–2.94), 0.099	1.78(0.75–4.25), 0.155	0.58(0.37–0.88), 0.007	1.75(1.14–2.69), 0.007
Dickinson et al. [18]	MS(136)	52(0.382)	68(0.500)	16(0.118)	172(0.632)	100(0.368)
	Controls(235)	86(0.366)	108(0.459)	41(0.175)	280(0.596)	190(0.404)
	OR(95% CI), p	1.07(0.68–1.70), 0.753	1.18(0.75–1.84), 0.453	0.63(0.32–1.22). 0.144	1.17(0.85–1.61), 0.325	0.86(0.62–1.18), 0.325
Smolders et al. [20]	MS(212)	83(0.391)	96(0.453)	33(0.156)	262(0.618)	162(0.382)
	Controls(289)	112(0.388)	138(0.477)	39(0.135)	362(0.626)	216(0.374)
	OR(95% CI), p	1.02(0.70–1.49), 0.928	0.91(0.63–1.31), 0.585	1.18(0.70–2.01), 0.514	0.97(0.74–1.26), 0.787	1.04(0.79–1.35), 0.787
Simon et al. [15]	MS(214)	86(0.402)	107(0.500)	21(0.098)	279(0.652)	149(0.348)
	Controls(428)	154(0.360)	205(0.479)	69(0.161)	513(0.599)	343(0.401)
	OR(95% CI), p	1.20(0.84–1.70), 0.300	1.09(0.77–1.53), 0.616	0.57(0.33–0.98), 0.030	1.25(0.98–1.61), 0.068	0.80(0.62–1.02), 0.068
Sioka et al. [21]	MS(69)	30(0.435)	30(0.435)	9(0.130)	90(0.652)	48(0.348)
	Controls(81)	33(0.408)	36(0.444)	12(0.148)	102(0.630)	60(0.370)
	OR(95% CI), p	1.12(0.55–2.26), 0.736	0.96(0.48–1.93), 0.906	0.86(0.31–2.39), 0.756	1.10(0.67–1.82), 0.686	0.91(0.55–1.50), 0.686
Agliardi et al. [22]	MS(641)	219(0.342)	308(0.480)	114(0.178)	746(0.582)	536(0.418)
	Controls(558)	220(0.394)	249(0.446)	89(0.160)	689(0.617)	427(0.383)
	OR(95% CI), p	0.80(0.63–1.02), 0.059	1.15(0.91–1.45), 0.236	1.14(0.83–1.56), 0.398	0.86(0.73–1.02), 0.077	1.16(0.98–1.37), 0.077
Cox et al. [19]	MS(727)	NA	NA	NA	843(0.580)	611(0.420)
	Controls(604)	NA	NA	NA	749(0.620)	459(0.380)
	OR(95% CI), p	–	–	–	0.85(0.72–0.99), 0.035	1.18(1.01–1.39), 0.035
Irízar et al. [23]	MS(136)	55(0.404)	70(0.515)	11(0.081)	180(0.662)	92(0.338)
	Controls(337)	145(0.430)	157(0.466)	35(0.140)	447(0.663)	227(0.337)
	OR(95% CI), p	0.90(0.59–1.38), 0.607	1.22(0.80–1.85), 0.337	0.76(0.35–1.61), 0.446	0.99(0.73–1.35), 0.966	1.01(0.74–1.37), 0.966
García-Martín et al.(Current study)	MS(303)	129(0.426)	129(0.426)	45(0.148)	387(0.639)	219(0.361)
	Controls(310)	131(0.423)	144(0.464)	35(0.113)	406(0.655)	214(0.345)
	OR(95% CI), p	1.01(0.73–1.41), 0.937	0.86(0.61–1.19), 0.335	1.37(0.83–2.26), 0.191	0.93(0.73–1.19), 0.552	1.07(0.84–1.37), 0.552
TOTAL SERIES	MS(2944)	821/1396(0.370)	1068/1149(0.482)	328/1889(0.148)	3553(0.603)	2335(0.397)
	Controls(3166)	1009/1553(0.394)	1183/1379(0.462)	370/2192(0.144)	3950(0.624)	2382(0.376)
	OR(95% CI), p	0.91(0.80–1.02), 0.095	1.08(0.97–1.22), 0.168	1.03(0.87–1.21), 0.730	0.92(0.85–0.99), 0.021	1.09(1.01–1.17), 0.021

## Discussion

The possible role of vitamin D in the pathogenesis of MS makes reasonable to analyse the possible relationship of *VDR* polymorphisms and gene allelic variants with the risk of MS. In fact, several studies found direct association of MS risk with the SNPs *VDR* rs2228570 [14]–[16] and rs731236 [19], respectively. Moreover, some studies showed modification of the MS risk related with the interaction of rs2228570 with vitamin D intake [15] or with *HLA-DR15* [24] or with the interaction of rs731236 with *HLA-DRB1* [19], [22], [23].

In the present study, we found no significant differences either in the frequencies of rs2228570 and rs731236 genotypes, or in the frequencies of the allelic variants of these polymorphisms in patients with MS. In addition the frequencies for the polymorphisms analyzed were neither related with the age at onset of MS, or with the evolutive type of MS. However, the present study has some limitations. First, the size of analyzed cohorts may not be sufficient for strict conclusions about VDR role in MS, and individual studies of small number of patients gave very contradictory results. Second, despite the sample size is adequate to detect an OR as small as 1.5, a more modest association would not be detected (this is a usual weakness of genetic association studies). Third, because the cohort study included MS patients with different degrees of severity, it is not adequate for the investigation of the influence of *VDR* genotypes on the disability or severity of MS (the ideal study for this purpose should include genotyping of patients with a recent diagnosis of MS with similar follow-up periods).

The results of the current study regarding rs2228570 are in agreement with those of other previous case-control studies [15]–[19], In contrast, Partridge et al. [14] reported a modest decrease in the risk for MS in homozygous for the minor allele, but the global minor allele frequencies of MS patients did not differ significantly between their MS patients and controls. As should be expected, the results of the meta-analysis, including the previous and the current study, confirmed the lack of association between rs2228570 and MS risk.

The results of the current study regarding rs731236 are in agreement with other 6 studies [14], [18], [20]–[23], while only three previous reports showed modification of MS risk related with this SNP. Tajouri et al. [16], in a study including 104 MS patients and 93 controls, reported increased risk for minor allele carriers with an odds-ratio of 1.75(1.14–2.69), although this result should be related with a higher minor allele frequency in MS patients than that described in other series. In contrast, the report by Simon et al. [15], which included 212 MS patients and 289 controls, showed a modest decrease in MS risk in homozygous for the minor allele, with an odds-ratio of 0.57(0.33–0.98), while the differences in the minor allele frequencies between MS patients and controls did not reach statistical significance. Finally, the report by Cox et al. [19], which included 727 MS patients and 604 controls, showed a modest decrease in MS risk for minor allele carriers, with and OR of 1.18(1.01–1.39), a finding that they also found when they analyzed the combined their case-control and trio family datasets.

The results of the meta-analysis of previous and current studies on *VDR* rs2228570 and rs731236 indicate a lack of association of rs2228570 and rs731236 with the risk for MS, in spite of the marginal statistical significance of the crude pooled values for rs731236 shown in Table 7. This apparent discrepancy is related to the use of the DerSimonian-Laird method for the calculation of the diagnostic OR. This method is more restrictive than the Mantel-Haenszel method that gives the same results as shown in Table 7 (P = 0.029).

In the present study, we confirmed the previously described association between HLADRB1*1501 (rs3135388) haplotype and the risk of developing MS. On the other hand, we did not find association between the interaction of rs2228570 and rs731236 SNPs with HLADRB1*1501 haplotype and MS risk. The comparison with other studies addressing this issue is difficult taking in account the data reported because the heterogeneity of both the methodology and the results:

- The first study, reported by Orton et al. [24] was not a case-control study, but a family based study, described a modest increased MS risk in HLA-DR15 negative subjects carrying the rs2228570T allele, and found no significant interaction between rs731236 and the risk for MS.
- Agliardi et al. [22], in a case-control including 187 MS patients and 197 controls HLA-DRB1*15-positive and 454 MS patients and 363 controls HLA-DRB1*15-negative, found a modest decrease in the risk for MS in HLA-DRB1*15-positive subjects carrying the rs731236TT genotype or the rs731236T allele. They also described lack of interaction between *HLA-DRB1* and rs2228570 and rs1989969 SNPs and the risk for MS.
- Cox et al. [19], using the combination of a case-control cohort including 727 MS patients and 604, and 1153 trio families(MS patient and both parents), found a trend towards increased risk for subjects carrying rs3135388AA and rs2228570CC genotypes, with an OR of 7.198(2.62–19.78), and for subjects carrying rs3135388 AA and rs731236CC genotypes, with an OR of 3.014(2.019–4.498), all of them compared with subjects with TT genotypes for both SNPs). However, data regarding genotype and allele distribution are not given.
- Irízar et al. [23] reported a non-significant trend towards increased risk for MS in HLA-DRB1*15-positive subjects carrying rs7975232A or rs731236T alleles in a case-control study which included 325 HLA-DRB1*15-positive subjects (96 MS patients and 229 controls) and 110 HLA-DRB1*15-negative subjects (34 MS and 76 controls) genotyped for rs7975232, and 392 HLA-DRB1*15-positive subjects (122 MS patients and 270 controls) and 43 HLA-DRB1*15-negative subjects (11 MS and 32 controls) genotyped for rs731236.

In summary, the results of the present study, taken together with those of the meta-analysis, suggest that rs2228570 and rs731236 genotype and allelic variants are not related with the risk for MS. In addition, although this study confirmed the association between rs3135388 SNP and the risk for MS, the previously suggested interaction between *HLADRB1* and the two studied SNPs in the *VDR* gene in the risk of developing MS was not confirmed.

## Supporting Information

Figure S1 **Prisma flow-chart for rs2228570** (**Fok1**) **polymorphism.**
(DOC)

Figure S2 **Prisma flow-chart for rs731236** (**Taq1**) **polymorphism.**
(DOC)

Table S1 **Prisma statement.**
(DOC)
