# Supplementary material for: Vitamin D3 Receptor ( VDR ) Gene rs2228570 (Fok1) and rs731236 (Taq1) Variants Are Not Associated with the Risk for Multiple Sclerosis: Results of a New Study and a Meta-Analysis
Source: PLoS One. 2013 Jun 20;8(6):e65487. doi: 10.1371/journal.pone.0065487 (PMC3688728; doi:10.1371/journal.pone.0065487)
Supplement: Figure S2 — Prisma flow-chart for rs731236 (Taq1) polymorphism. (DOC) [file pone.0065487.s002.doc]

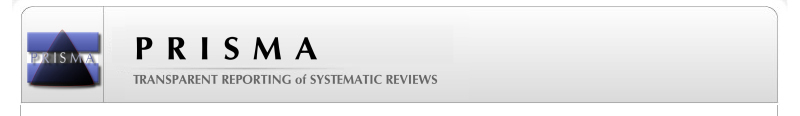
**PRISMA 2009 Flow Diagram**

**Screening**

**Included**

**Eligibility**

**Identification**

Records identified through database searching
(n =9 )

Additional records identified through other sources
(n = 0 )

Records after duplicates removed
(n =9 )

Records screened
(n = 9 )

Records excluded
(n =0 )

Full-text articles assessed for eligibility
(n = 9 )

Full-text articles excluded, with reasons
(n = 0 )

Studies included in qualitative synthesis
(n = 9 )

Studies included in quantitative synthesis (meta-analysis)
(n = 9 )

**rs731236 (Taq1)** *

* Excluding current study
